# Supplementary material for: Assessing Pragmatic Skills in People with Intellectual Disabilities
Source: Behav Sci (Basel). 2025 Feb 27;15(3):281. doi: 10.3390/bs15030281 (PMC11939783; doi:10.3390/bs15030281)
Supplement: Supplementary file 1 [file behavsci-15-00281-s001.zip › Table S2 Selected articles, objective and design.pdf]

Table S2: Selected articles, objective and design

	Selected articles	Design	Objetive
1	Alfieri, P. et al. (2017).	Quasi-experimental	Assess whether linguistic and sociocommunicative skills are in line with their age.
2	Angell, M. E. et al. (2008).	Case series study	Evaluate the application of a systematic instructional program.
3	Courchesne, V. et al. (2020).	Case study	Provide a psychological example of how an autistic adolescent manages their interests and strengths.
4	del Hoyo Soriano, L. et al. (2018).	Quasi-experimental	Compare the phenotypes of people with DS and SFX through the evaluation of various domains of communication.
5	Diez-Itza, E. et al. (2016).	Phenomenological Study	Evaluate the coherence and cohesion of the narrative competence of adults with WS.
6	Diken, Ö. (2014).	Quasi-experimental	Exploring the pragmatic language skills of children with TD (ASD and ID) in Türkiye.
7	Đorđević, M. et al. (2014).	Systematic review	Analyze research whose focus was evaluating paralinguistic elements in adults with ID.
8	Hagan, L. et al. (2014).	Case study	To evaluate the impact of a speaking-generating instrument on the communicative and pragmatic skills of a non-verbal woman with moderate ID.
9	Hoffmann, A. et al. (2013).	Quasi-experimental	Compare pragmatic skills in individuals with WS.
10	Iacono, T. A. et al. (1996).	Quasi-experimental	Compare a structured condition versus an unstructured condition, the frequency of requests and comments, and identify which modalities children use.
11	Jenkins, C. et al. (1998).	Quasi-experimental	Compare the prelinguistic skills of young children with DS, children with TD, and other children with learning disabilities.
12	John, A. E. et al. (2012).	Phenomenological Study	To evaluate the temporal stability of the different pragmatic skills of a group of children with WS.
13	Klusek, J. et al. (2014).	Quasi-experimental	Determine if the presence of ASD can be attributed to the pragmatic difficulties of people with SFX.
14	Martin, G. E. et al. (2013).	Quasi-experimental	Document similarities and differences, establish the developmental trajectory in different linguistic domains, and determine the influence of autism on SFX
15	McAtee, M. et al. (2004).	Study of psychometric properties	Develop a contextual assessment inventory and evaluate some of the psychometric properties of the inventory.

Table S2: Selected articles, objective and design

16	Owen, M. S. et al. (1994).	Phenomenological Study	Analyze communicative intention and frequency among adults with developmental disabilities and its residential staff
17	Shilc, M. et al. (2017).	Phenomenological Study	Study the differences in narrative skills and observe the lexical, grammatical and structural characteristics of speech.
18	Van Den Heuvel, E. et al. (2016).	Quasi-experimental	To compare expressive and receptive language and two pragmatic skills between children with WS and children with idiopathic ID.
19	Van Den Heuvel, E., et al. (2018).	Quasi-experimental	Compare different expressive and receptive language skills between normotypical children and children with different disorders.
20	Wellnitz, S. A. et al. (2021).	Study of psychometric properties	Validate and improve the CCC-2 among the different diagnoses, making it more concise, simplifying it and revising it.
