# Supplementary material for: Assessing Pragmatic Skills in People with Intellectual Disabilities
Source: Behav Sci (Basel). 2025 Feb 27;15(3):281. doi: 10.3390/bs15030281 (PMC11939783; doi:10.3390/bs15030281)
Supplement: Supplementary file 1 [file behavsci-15-00281-s001.zip › Table S3 Evaluation instruments of the selected studies..pdf]

Table S3 Evaluation instruments of the selected studies.

	Selected articles	Pragmatic evaluation tools	Components of Pragmatics	Other cognitive or language tests	Figure
1	Alfieri, P. et al. (2017).	VABS	VABS Communication Mastery	Leiter-R, PPVT-r, PVCL, BNT	Parents
2	Angell, M. E. et al. (2008).	OD	1) Mandatory turn taking.<br>2) Non-mandatory turn taking.<br>3) Ask focused questions to your partner.<br>4) Converse with appropriate eye contact.<br>5) Use an appropriate tone of voice in conversational speech.		
3	Courchesne, V. et al. (2020).	VABS-II	1) Adaptive behaviors<br>2) Communication<br>3) Daily living skills<br>4) Socialization	RPM, WISC-V, CELF-CDN-F, WIAT-II, EOWPVT-IV, EVIP, LE Vol du PC, BALE	1 (Mother)
4	del Hoyo Soriano, L. et al. (2018).	1) CCC-2<br>2) SALT	1) Loquacity<br>2) Unintelligibility<br>3) Disfluency<br>4) Lexical diversity<br>5) Syntactic complexity<br>6) Inappropriate initiation<br>7) Stereotyped language<br>8) Use of context and<br>9) Non-verbal communication	Leiter-R, PPVT-III, CASL-EVT, CASL-SC, FBT, CBCL/6-18	2 (Parents)
5	Diez-Itza, E. et al. (2016).	1) OD<br>2) Recount of the sequential order of events.<br>3) Block Design subtest.<br>4) Average use of discourse markers.<br>5) *"Frog Goes To Dinner"*.	Coherence and cohesion of pragmatic competence.	PPVT, WAIS-III	

6	Diken, Ö. (2014).	TV-PLSI	1) Stereotypical behaviors, communication and social interaction<br>2) Classroom interaction skills, Social and personal interaction skills	TV-GARS-2	Special education school teachers
7	Đorđević, M. et al. (2014).	1) Electrophysiological study<br>2) DANVA-2<br>3) FAB<br>4) TFER<br>5) SRAI<br>6) Photos with facial expression	Recognize emotional prosody and emotions	1) Intelligence test<br>2) Visual discrimination	
8	Hagan, L. et al. (2014).	1) Pragmatic checklist designed by researchers<br>2) OD	1) Beginning of communicative interaction.<br>2) Presentation of a specific topic.<br>3) Respect for taking turns.<br>4) Thematic continuity.<br>5) Request for clarification.<br>6) Appropriate eye contact.<br>7) Use of gestures and facial expressions.		1-day center member and 1 residential center person
9	Hoffmann, A. et al. (2013).	1) TOPL-2<br>2) CCC-2	1) TOPL-2: physical context, audience, theme, purpose, visual and gestural cues, abstractions and pragmatic evaluation<br>2) CCC-2: pragmatics, syntax, morphology, semantics and speech.	KBIT-2	Parents
10	Iacono, T. A. et al. (1996).	1) VABS<br>2) MCDI<br>3) OD	1) Requests and comments<br>2) Communicative acts	RDLS-R	1<br>(Mother)
11	Jenkins, C. et al. (1998).	1) "Pragmatic profile of early communication skills"<br>2) OD	Pre-linguistic skills	DLS	1)Father<br>2)Mother
12	John, A. E. et al. (2012).	1) OD based on the game<br>2) Conversation with a family researcher.	1) Number of sentences that the child makes and that serve to adapt to the context.<br>2) Questions in which the child made eye contact with the interlocutor.<br>3) Eye contact that the child makes while producing the statements.	DAS, EVT	Mothers

13	Klusek, J. et al. (2014).	1) CASL–PJ<br>2) PRS–SA	1) Communicative intention, turn taking, emotional expression and pragmatic adaptation<br>2) Verbosity, social appropriateness, scripting, redundancy, failure to initiate topics, inappropriate turns, eye contact and communicative gestures.	Leiter-R, PPVT-III, EVT, ADOS	
14	Martin, G. E. et al. (2013).	CASL-PJ	Knowledge of appropriate language for various social situations.	Leieter-R, CASL-Antonyms, CASL-SC, ADOS	
15	McAtee, M. et al. (2004).	1) Checklist CAI<br>2)OD	1) Negative interactions and disagreements.<br>2) Factors related to tasks and factors related to daily routines.<br>3) Uncomfortable environment and changes in the environment.		2<br>(Support staff)
16	Owen, M. S. et al. (1994).	OD	Speech acts, communicative initiative, speaking turns and type of statements		1 H/1M<br>(Support staff)
17	Shilc, M. et al. (2017).	*The Storytelling Test: Illustrations of The Frog King*	Vocabulary, grammatical structure and structure of the content of the stories.		
18	Van Den Heuvel, E. et al. (2016).	1) APT<br>2) ITS-APT<br>3) RTNA-BST<br>4) Coding of statements into five categories<br>5) ITS-BST	1) Perspective-taking ability.<br>2) Quality and quantity of information transfer.<br>3) Manner and relevance of information transfer: coding of the statements into five categories.<br>4) Narrative capacity.	WPPSI-III-NL, SON, PPVT-III-NL, CELF-P2-NL, CELF-4-NL	
19	Van Den Heuvel, E., et al. (2018).	FS (CELF-5)	Ability to interpret and use contextual information	WPPSI-III-NL, SON R6-40, PPVT-III-NL, CELF-4-NL, CELF-P2-NL, RS	
20	Wellnitz, S. A. et al. (2021).	CCC-R, CCC-2	It is made up of 39 items, instead of the 70 items that make up the original checklist.	CBCL, SRS, SQC, Demographics data sheet	

ABBREVIATIONS PRAGMATIC EVALUATION TOOLS: VABS: Vineland Adaptive Behavior Scales, FS: Subtest Formulating Sentences) Clinical Assessment of Language Fundamentals-Fifth Edition (CELF-5), CCC-R: Revised Children's Communication Checklist-2, CCC-2: Children's Communication Checklist-2, CASL-PJ: Comprehensive Assessment of Spoken Language – Pragmatic Judgement, PRS–SA: Pragmatic Rating Scale—School Age, ADOS: Autism Diagnostic Observational Schedule, SALT: Systematic Analysis of Language Transcripts, APT: Action Picture Test, ITS-APT: Information Transfer Score, RTNA-BST: Renfrew Language Scales Dutch Adaptation - Bus Story Test, ITS-BST: "Information Transfer Score", MCDI: Macarthur Communicative Developmental Inventory, TV-PLSI: Turkish Version of the Pragmatic Language Skills Inventory, OD: Observación Directa, CAI: Contextual Assessment Inventory, TOPL-2:

Test of Pragmatic Language-2, DANVA-2: Diagnostic Analysis of Nonverbal Accurcy Scale-2, FAB: Florida Affect Battery, TFER: Test of Facial Emotion Recognition, SRAI: Self-Report Anger Inventory.

ABBREVIATIONS OTHER COGNITIVE AND LANGUAGE TESTS: RPM: Raven's Standard Progressive Matrices, WISC-V: Wechsler Intelligence Scales for Children – Fifth Edition, CELF-CDN-F: Clinical Evaluation of Language Fundamentals – French Canadian Version, WIAT-II: Wechsler Individual Achievement Test—Second Edition, EOWPVT-IV: Expressive One-Word Picture Vocabulary Test—Fourth Edition, EVIP: Échelle de Vocabulaire en Images Peabody (French Version of the Peabody Picture Vocabulary Test), BALE: Batterie Analytique du Langage Écrit, WPPSI–III–NL: Wechsler Preschool and Primary Scale of Intelligence–III, Dutch Edition, SON R6–40: Categories and Analogies subtests from the Snijders-Oomen Nonverbal Intelligence Test Revised age 6–40, PPVT–III–NL: Receptive Vocabulary Age Equivalents (RVAE) of the Dutch edition of the Peabody Picture Vocabulary Test, CELF–4–NL: Dutch adaptation of the Clinical Evaluation of Language Fundamentals–Fourth Edition, CELF-P2-NL: Clinical Evaluation of Language Fundamentals-Preschool–Second Edition, RS: The Recalling Sentences, CBCL: Child Behavior Checklist, SRS: Social Responsiveness Scale, SCQ: Social Communication Questionnaire, Leiter-R: Leiter International Performance Scale-Revised, ADOS: Autism Diagnostic Observation Schedule, CASL-Antonyms: Comprehensive Assessment of Spoken Language – Antonyms, CASL-SC: Comprehensive Assessment of Spoken Language – Syntax Construction, PPVT-III: Peabody Picture Vocabulary Test – Third Edition, CASL-EVP: Comprehensive Assessment of Spoken Language - Expressive Vocabulary Test, FBT: False Belief Task, CBCL/6-18: Child Behavior Checklist, Ages 6–18, WAIS-III: Wechsler Adult Intelligence Scale, RDLS-R: Reynell Developmental Language Scales – Revised, TV-GARS-2: Turkish Version of the Gilliam Autism Rating Scale-2, DLS: Derbyshire Language Scheme, KBIT-2: The Kaufman Brief Intelligence Test, Second Edition, DAS: Differential Ability Scales, PPVT-r: Peabody Picture Vocabulary Test – Revised, PVCL: Prove di Valutazione delle Competenze Linguistiche, BNT: Boston Naming Test.
